# Exercise Types: Physical Activity Mitigates Cardiac Aging and Enhances Mitochondrial Function via PKG-STAT3-Opa1 Axis

**DOI:** 10.14336/AD.2024.0959

**Published:** 2025-10-30

**Authors:** Reka Szekeres, Daniel Priksz, Mariann Bombicz, Beata Pelles-Tasko, Anna Szilagyi, Brigitta Bernat, Aniko Posa, Balazs Varga, Rudolf Gesztelyi, Sandor Somodi, Zoltan Szabo, Zoltan Szilvassy, Bela Juhasz

**Affiliations:** ^1^Department of Pharmacology and Pharmacotherapy, Faculty of General Medicine, University of Debrecen, Debrecen, Hungary.; ^2^University of Debrecen, Doctoral School of Nutrition and Food Sciences, Debrecen, Hungary.; ^3^Department of Oral Biology and Experimental Dental Research, Faculty of Dentistry, University of Szeged, Szeged, Hungary.; ^4^Department of Emergency Medicine, Faculty of General Medicine, University of Debrecen, Debrecen, Hungary

**Keywords:** cardiac aging, Opa1, ATPS, exercise, mitochondria

## Abstract

Although age-related deterioration of the cardiac function is a well-studied area of research, the interventions and their molecular pathways have not yet been fully identified. Since physical activity is a powerful preventive measure against cardiac aging, our study compared the effects of long-term voluntary and forced physical activity with a sedentary group, utilizing an aging rat model characterized by mitochondrial dysfunction that contributes to age-related cardiovascular diseases. Four experimental groups were created: (I) young controls (12-week-old); (II) 18-month-old aged sedentary rats; (III) aged group with free access to running wheels for 6 months; (IV) aged rats subjected to forced physical activity for 6 months. At the endpoint of the study, the aged animals were two years old. The aged sedentary rats exhibited increased Tei-index, LA/Ao and E/e’ ratios as well as decreased e’/a’ ratio and lengthened DecT and IVRT, higher perivascular fibrosis ratio and reduced myocardial PKG, STAT3 and Opa1 protein expression, along with decreased ATP synthase (ATPS) activity in comparison to the young controls. In terms of echocardiographic parameters and perivascular fibrosis, the forced running provided more substantial benefits than the voluntary activity demonstrated by decreased Tei-index, E/e’ ratio, increased e’/a’ ratio and reduced DecT and IVRT. Forced exercise was strongly associated with elevated myocardial expression of PKG, STAT3 and Opa1 proteins and, moreover, the ATPS activity was restored only in the forced running rats. In conclusion, forced but not voluntary exercise has significant protective effects on age-associated diastolic dysfunction by upregulating PKG-STAT3-Opa1 axis and thereby enhancing ATPS activity.

## INTRODUCTION

Age-related heart failure (HF) is one of the greatest matters of increasing concern confronting global health care nowadays [1]. The rising prevalence of aging-associated hypertension, diabetes mellitus and coronary diseases may contribute to the higher incidence of heart failure in the elderly [2]. On the other hand, extensive evidence suggests that - even in the absence of any pathological conditions - cardiac senescence unfolds as a slowly progressive process characterized by structural changes and functional declines of the myocardium [3]. Moreover, advancing age is accompanied by a sterile, low-grade chronic inflammation called “inflammaging”, which is a long-term result of the sustained activation of the innate immune system and contributes to the pathogenesis of several age-related diseases [4, 5].

Age-related left ventricle (LV) hypertrophy and cardiac fibrosis cause increased ventricular stiffness resulting in delayed myocardial relaxation, thus impaired LV filling and elevated LV filling pressure [6]. These alterations of the chambers are concomitant with reduced exercise tolerance and atrial changes as well, increasing the risk of atrial fibrillation as left atrial hypertrophy and dilation heighten the susceptibility to such arrhythmias [3]. The cumulative effect of these events predisposes older individuals to develop age-related diastolic dysfunction, serving as a precursor of the symptomatic diastolic heart failure [7].

Several studies have explored factors influencing cardiac aging, revealing that the aging process in cardiomyocytes is often accompanied by a decline in mitochondrial function [8]. Impairments in the mitochondrial quality control (MQC) processes such as mitochondrial fusion-fission dynamics, mitophagy and biogenesis exhibit in diminished performance of the aged heart [9, 10]. The results of numerous research leave no room for doubt regarding the fact that the imbalance between mitochondrial fusion and fission is one of the key pathological mechanisms of cardiac aging [11]. Furthermore, during myocardial senescence, the increased oxidative stress contributes to mitochondrial dysfunction, which subsequently generates increased amount of reactive oxygen species (ROS) leading to a downward spiral in the cardiac function [12].

**Figure 1.**
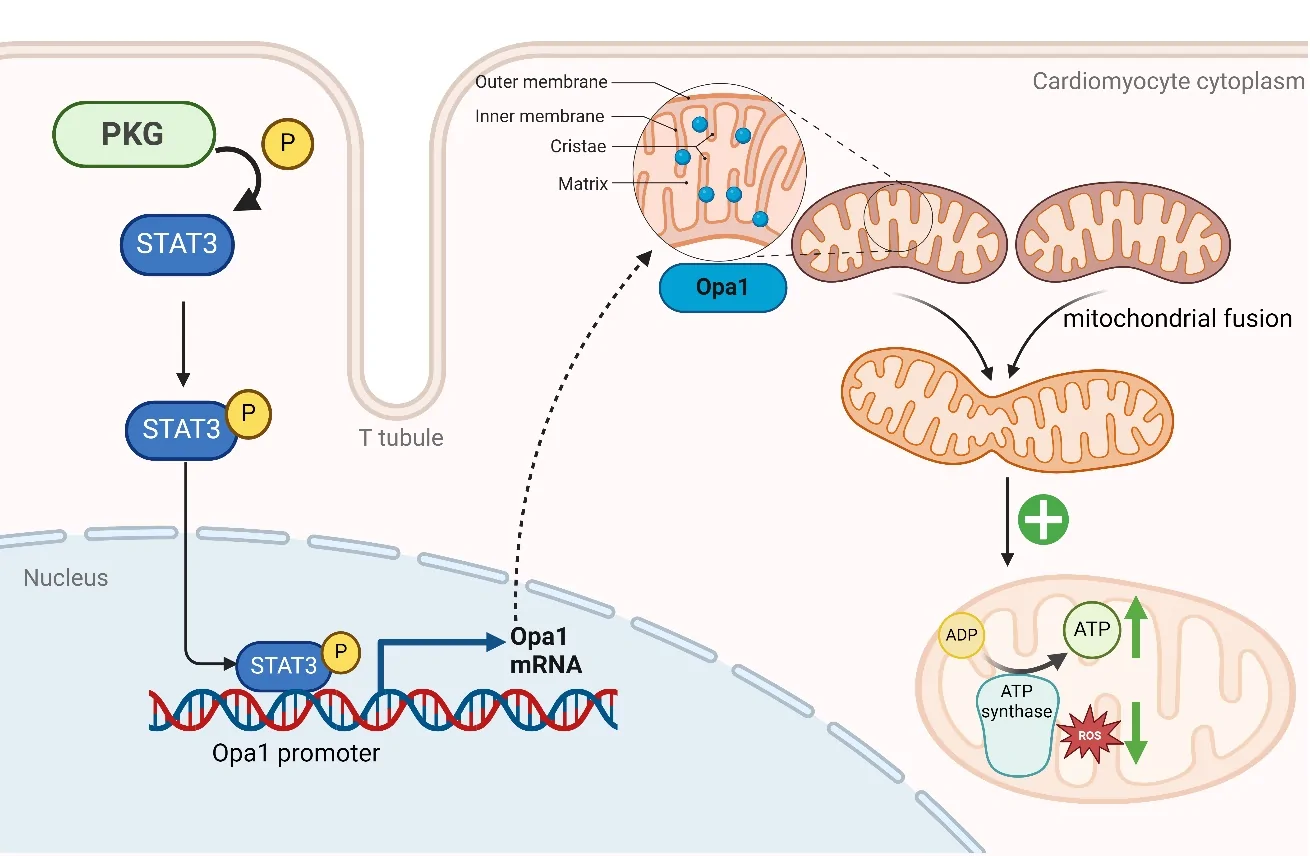
**The PKG-STAT3-Opa1 axis**. Firstly, PKG phosphorylates STAT3, which then translocates into the nucleus and promotes the transcription of Opa1. Secondly, Opa1 fuses the inner membranes of the mitochondria, and, finally, mitochondrial fusion enhances ATP generation. PKG: protein kinase G, STAT3: signal transducer and activator of transcription 3, Opa1: optic atrophy 1. Adapted from “Cardiomyocyte Cell (Layout)”, by BioRender.com (2024). Retrieved from https://app.biorender.com/biorender-templates.

Mitochondrial fusion is recognized for its beneficial effects, as it significantly enhances ATP generation along with the inhibition of mitochondria-derived ROS production [9, 13]. The PKG-STAT3-Opa1 pathway has been linked to cellular responses to oxidative stress, as well as the regulation of the mitochondrial fusion in the cardiomyocytes [14]. PKG, an essential mediator in cardioprotection [15] subsequently induces STAT3 activation, which translocates into the nucleus and promotes Opa1 transcription [13, 16]. Then, Opa1 fuses the inner membranes of the adjacent mitochondria, moreover, it controls cristae shaping and maintains mitochondrial energetics (Figure 1) [17, 18].

It is well documented that living an active lifestyle reduces the risk of CVD events and cardiovascular mortality [19]. Regular physical activity exerts moderating effects on dyslipidaemia, blood pressure and resting heart rate, thereby conferring cardioprotective advantages [20, 21]. Additionally, exercise attenuates chronic inflammation and amplifies mitochondrial capacity for oxidative phosphorylation (OXPHOS) and ATP synthesis [22, 23]. When utilizing animal models, it is essential to compare different exercise interventions and identify their molecular mechanisms to advance the translation of findings from animal studies to clinical applications.

The principal objective of this study was to explore whether the long-term voluntary or forced exercise regimen initiated at older age has more protective effect on cardiac aging. Another purpose of the investigation was to examine the relationship between aging and physical activity and the mitochondrial fusion regulator PKG-STAT3-Opa1 signaling pathway.

## MATERIALS AND METHODS

### Animals

In the current study 36 aged (18-month-old) and 9 young (12-week-old) male Wistar rats were used. All the experimental methods were approved by the University of Debrecen Committee of Animal Welfare (3/2022/DEMÁB) and complied with the ARRIVE guidelines [24]. The rats were purchased from Charles River Laboratories International, Inc. (Wilmington, MA, USA) and received humane care in accordance with the "Principles of Laboratory Animal Care" by EU Directive 2010/63/EU. All animals were kept under controlled conditions (2 rats per cage) at a temperature of 24 °C, but the 12:12 hour light/dark (L/D) cycle was reversed as rats are nocturnal. The reversal of the L/D cycle allowed us to carry out the exercise sessions during the awake phase of the rats and the normal working hours for the staff.

### Experimental Protocol

After two weeks of acclimatization, 4 experimental groups were formed: (I) young control rats (n = 9); (II) aged animals with sedentary lifestyle (n = 12); (III) aged rats representing voluntary exercise (n = 12); (IV) aged rats demonstrating forced physical activity (n = 12). Animals from the voluntary exercise model were housed in wheel-installed cages (Lafayette Instrument Company, Inc., North Lafayette, IN, USA) and had free access to the wheels 24 h a day according to their needs, for six months. Forced running was performed using Walking Wheel System for Rats (Lafayette Instrument Company, Inc., North Lafayette, IN, USA), an apparatus with electronically controlled motorized wheel drive. During the six months of the forced running, rats were placed into the running wheels six days a week and both the time and the speed of the exercise were gradually increased depending on the training status of the animals. The term forced physical activity refers to a regulated, moderate-intensity exercise performed for a longer time than the voluntary activity. Before the experiment the animals were conditioned at low speeds and durations to increase their capability and experience and to recognize the rats not suitable for the forced running.

**Figure 2.**
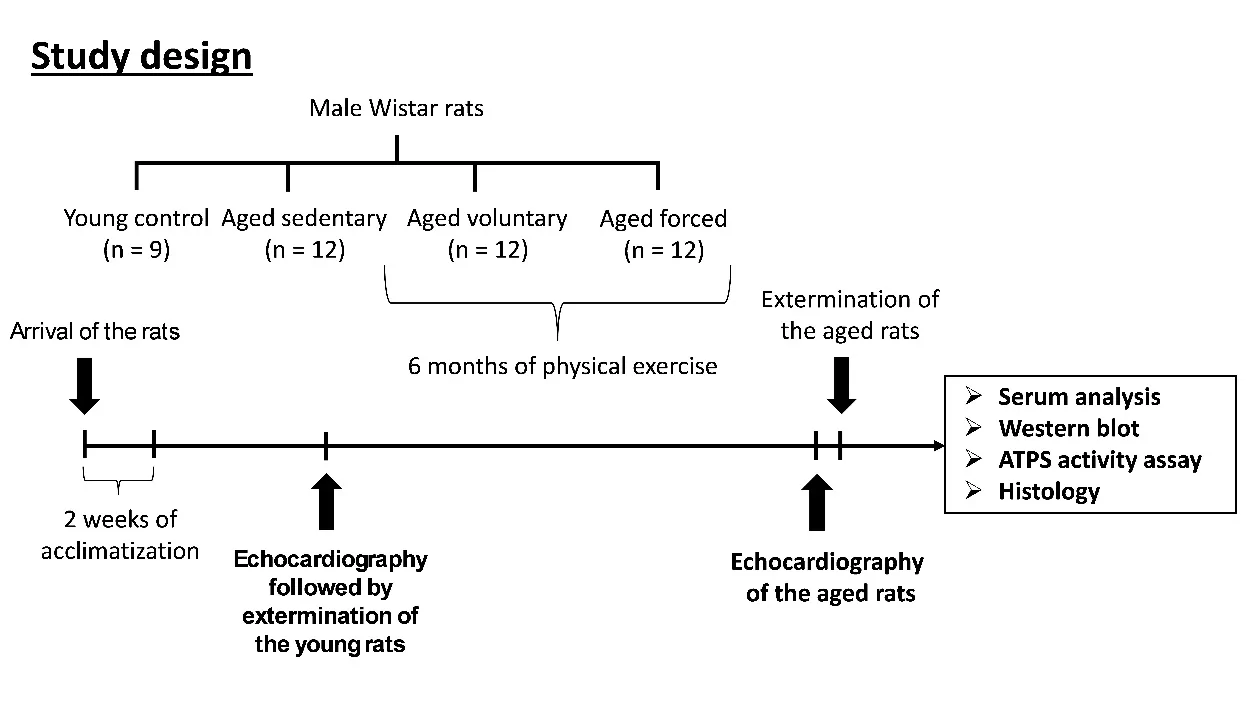
**Flowchart of the study**. After the 2-week-long adaptation period 4 experimental groups were created: (1) young control group (n = 9); (2) aged rats living physically inactive lifestyle (n = 12); (3) aged group with free access to the wheels inserted in their cage for 6 months (n = 12); (4) aged rats with forced exercise for 6 months (n = 12). In the case of all rats, after the echocardiographic measurements, humane euthanization was carried out, followed by several experimental procedures: serum analysis, Western blot, ATP synthase activity microplate assay and histological staining.

As shown in Figure 2, at the end of the six months the echocardiographic measurements of the aged rats were carried out. After this procedure thoracotomy was conducted under deep anaesthesia (100/10 mg/kg ketamine/xylazine, intramuscular injection), followed by blood sample collection via cardiac puncture. Furthermore, the left ventricle tissue samples were either frozen in liquid nitrogen and then stored at -80 °C for Western blot method and ATP synthase activity assay or fixed in 10% formalin solution for later histological analysis. The young counterparts were euthanized at the age of 3 months.

### Forced Exercise Protocol

The aged rats from the forced exercise group were subjected to forced wheel running 6 days per week, with a resting day on Sundays, for 6 months. Firstly, rats underwent a habituation protocol for one week (speed: 5 m/min; duration: 5 min) to reduce stress responses and establish a more homogenous starting point for all running animals. The speed was then gradually increased by 0.5 m/min until a speed of 13 m/min was achieved. Then the duration was increased incrementally by 1 minute each time until 20 minutes was reached. In contrast to the treadmill running protocols, no electric shock was applied, as the moving wheel itself encourages the aged rat to exercise, and electric stress is not recommended in aging studies.

### Chemicals

All reagents and solutions used for both the protein extraction and the Western blot method were purchased from Sigma-Aldrich-Merck KGaA (Darmstadt, Germany) and Abcam Plc. (Cambridge, UK). ATP synthase assay was acquired from Abcam Plc. (Cambridge, UK). For the histological staining the Masson’s trichrome kit was obtained from BioGnost Ltd. (Zagreb, Croatia).

### Transthoracic Echocardiography

At the endpoint of the study, echocardiography as an imaging modality was carried out using a Vivid E9 ultrasound machine (GE HealthCare Technologies, Inc., Chicago, IL, USA) with a high-frequency probe (12S-D). The cardiac ultrasound was performed under ketamine-xylazine anaesthesia (50/5 mg/kg, intramuscular injection), followed by chest hair removal. The animals were placed into dorsal and lateral decubitus positions with continuous ECG monitoring. The images were recorded from parasternal long- (PLAX) and parasternal short axis (PSAX), as well as from apical 4- and 5-chamber views in accordance with the recommendations of the American Society of Echocardiography [25]. The diameters of the aortic root (Ao, mm) and the left atria (LA, mm) were measured in M-mode followed by the offline calculation of the LA/Ao ratio. The left ventricle (LV) internal diameter in systole (LVIDs, mm) and in diastole (LVIDd, mm), the anterior and the posterior wall thickness of the LV in systole and diastole (LVAWs,d; LVPWs,d, mm), the ejection fraction (EF, %) and the fractional shortening (FS, %) were determined from M-mode recordings at the mid-papillary level. The diastolic function of the LV was assessed by pulsed wave (PW) Doppler and Tissue Doppler imaging (TDI) techniques from apical 4-chamber views. The E (early) and A (atrial) transmitral flow velocities (mm/s), the E/A ratio and the deceleration time of the E wave (DecT, ms) were defined from Doppler imaging. TDI measurements were conducted to record the motion of the myocardial tissue at both the septal and the lateral annulus: the systolic myocardial velocity (s’, mm/s), the early (e’, mm/s) and the atrial (a’, mm/s) diastolic myocardial velocities, the mitral valve closure to opening time (MCOT, ms), the ejection time (ET, ms), the isovolumetric contraction (IVCT, ms) and the isovolumetric relaxation time (IVRT, ms) were determined. Moreover, E/e’ (proposed as a non-invasive measure of left ventricular filling pressure), e’/a’ and stroke volume were also calculated. The Myocardial Performance Index (MPI), also known as Tei-index, was defined as the sum of the isovolumetric contraction and relaxation times divided by the ejection time. The parameters of the left ventricle outflow tract (LVOT) were obtained: the maximal and mean pressure gradients (PG, mmHg) and the velocities (Vel, mm/s) were evaluated from apical 5-chamber views. The echocardiograms were stored on a portable hard drive and later analyzed by a blinded reader using EchoPAC PC software (ver. 112, GE HealthCare Technologies, Inc., Chicago, IL, USA). All values were averaged over three consecutive cycles.

### Blood Sample Collection

After thoracotomy, blood samples were collected directly from the left ventricles of each rat into BD Vacutainer SST II Advance Tubes (BD Vacutainer, Bergen County, NJ, USA), using 25G syringe needles. All the serum parameters were determined by computerized laboratory analyzers (Roche Diagnostics GmbH, Mannheim, Germany) in the Department of Laboratory Medicine at the University of Debrecen. The lipid profile of the animals was analyzed by measuring the serum levels of total cholesterol, low-density lipoprotein cholesterol (LDL-C), high-density lipoprotein cholesterol (HDL-C) and triglyceride (TG). The atherogenic index of plasma (AIP=TG/HDL-C) was also calculated. Furthermore, to assess the function of the liver, aspartate transaminase (AST) and alanine transaminase (ALT) levels were measured and other parameters like glucose and C-reactive protein (CRP) levels were determined as well.

### Western blot

Western blot technique was used to identify the expression levels of the proteins extracted from the left ventricle tissue samples, as described previously [26]. In brief, 300 mg of the deeply frozen myocardial samples were homogenized in 800 µl buffer solution containing 25 mM Tris, 25 mM NaCl, 1 mM Na-orthovanadate, 10 mM NaF, 10 mM Na-pyrophosphate, 10 nM okadaic acid, 0.5 mM EDTA, 1 mM PMSF, protease inhibitor cocktail and distilled water (all from Sigma-Aldrich-Merck KGaA, Darmstadt, Germany). After series of centrifugation and aspiration of the resulting supernatants, cytosol- and mitochondria-containing fractions and nuclear extracts were separated. The total protein concentration of the samples was quantified using the BCA method (QuantiPro BCA Assay Kit, Sigma-Aldrich-Merk KGaA, Darmstadt, Germany). Finally, 50 μL of the samples of known protein content were mixed with Laemmli buffer (Sigma-Aldrich-Merck KGaA, Darmstadt, Germany) and the remaining quantities were stored at -80 °C.

After the tissue homogenization procedure, the samples diluted with Laemmli buffer were subjected to SDS-polyacrylamide gel electrophoresis (SDS-PAGE) (12% gel, 200 V). Then, the separated proteins were transferred onto a PVDF membrane (Cytiva, Global Life Sciences Solutions USA LLC, Marlborough, MA, USA) in a transfer buffer for 90 min at 25 V. 5% BSA solution was used for blocking the non-specific binding sites of the membranes (60 min) followed by the overnight incubation of the blots with the primary antibodies at 4 °C. The following antibodies were used: anti-glyceraldehyde-3-phosphate-dehydrogenase (GAPDH, as a housekeeping protein; Cat.No: G8795, Sigma-Aldrich-Merck KGaA, Darmstadt, Germany); anti-histone H3 (as a housekeeping protein; Cat.No: ab176842, Abcam Plc., Cambridge, UK); anti-protein kinase G (PKG, Cat.No: ab110124, Abcam Plc., Cambridge, UK); anti-signal transducer and activator of transcription 3 (STAT3, Cat.No: ab109085, Abcam Plc., Cambridge, UK); anti-optic atrophy 1 (Opa1, Cat.No: ab42364, Abcam Plc., Cambridge, UK); anti-ATP-synthase (ATPS, Cat.No: ab181243, Abcam Plc., Cambridge, UK). The next day, after washing the membranes with TBS-T for 3x10 minutes, horseradish-peroxidase (HRP)-conjugated anti-mouse or anti-rabbit secondary antibodies were applied for protein detection. The visualization of the protein bands was carried out using enhanced chemiluminescent substrate (Amersham™ ECL, Cytiva, Global Life Sciences Solutions USA LLC, Marlborough, MA, USA) and LiCor C-Digit® blot scanner (LI-COR Inc., Lincoln, NE, USA). The scanned Western blot images were analyzed with Image Studio Digits ver. 5.2. software (LI-COR Inc., Lincoln, NE, USA) during which normalization to the background and standardization to a housekeeping protein (GAPDH or Histon H3) were performed. In the case of all proteins, the average value of three independent experiments was used to perform statistical analysis (n = 3 per group).

### ATP Synthase Activity Microplate Assay

ATP synthase (also called Complex V) activity from LV tissue homogenates was detected by using the ATP Synthase Specific Activity Microplate Assay kit (ab109716, Abcam Plc., Cambridge, UK), according to the manufacturer’s instructions. Firstly, the enzyme was immunocaptured within the wells of the microplate. Then, its activity was measured as the rate of the ATP hydrolysis that is ultimately coupled to the oxidation of NADH to NAD^+^. The decrease in absorbance at 340 nm was monitored using a Varioskan LUX Multimode Microplate Reader (Thermo Fisher Scientific Inc., Waltham, MA, USA).

### Histology

To examine the correlation between tissue structure and function, LV samples were fixed in 10% neutral buffered formalin (pH = 7.4) for 24 hours. Next day, the samples were washed in water for 1 hour and then stored in 70% ethanol until further processes. To dehydrate the tissues the specimens were immersed in a series of more concentrated ethanol solutions until pure alcohol was reached. This process was followed by clearing with xylene, then paraffin wax was used as embedding material. Finally, from the paraffin-embedded blocks, 5 μm thick slices were sectioned and then stained with Masson’s trichrome, based on the protocol provided by the manufacturer (BioGnost Ltd., Zagreb, Croatia). The perivascular fibrosis ratio (PFR) was calculated as follows: the fibrotic area surrounding the vessel wall divided by the total vessel area. In each rat 5 randomly selected coronary vessels with a lumen diameter of over 50 µm were selected (n = 5 per group). The morphometric measurements were carried out under a magnification of 10 × using Nikon NIS-Elements BR (Ver5.41.00) software (Nikon Corp., Tokio, Japan).

### Statistical Analysis

The statistical analysis was carried out using GraphPad Prism software for Windows, version 8.00 (GraphPad Software Inc., La Jolla, CA, USA). Gaussian distribution of the data was evaluated by Shapiro-Wilk normality test. Then, datasets of the young control and aged sedentary groups were compared using unpaired t-test and one-way analysis of variance (ANOVA) followed by Tukey’s post hoc test was performed to determine the differences between the aged experimental groups. If the normality test was not passed, Kruskal-Wallis test followed by Dunn’s post-test was used for data analysis. Results were considered statistically significant when p < 0.05. All data are expressed as mean ± standard error of the mean (SEM).

**Table 1:** Results of the serum analysis.

Serum parameter	Young control	Aged sedentary	Aged voluntary	Aged forced
**Total cholesterol (mmol/L)**	1.843 ± 0.059	3.025 ± 0.137****	3.15 ± 0.171	3.156 ± 0.296
**LDL-C (mmol/L)**	0.3 ± 0.014	0.678 ± 0.035****	0.706 ± 0.059	0.621 ± 0.076
**HDL-C (mmol/L)**	1.266 ± 0.041	1.939 ± 0.109****	1.965 ± 0.118	2.039 ± 0.188
**Triglyceride (mmol/L)**	0.911 ± 0.074	1.066 ± 0.085	1.152 ± 0.078	1.21 ± 0.166
**Atherogenic index of plasma (AIP)**	0.718 ± 0.050	0.592 ± 0.085	0.619 ± 0.075	0.607 ± 0.071
**AST (GOT) (U/L)**	129.3 ± 7.875	200.3 ± 12.78****	172.1 ± 17.25	178.7 ± 13.66
**ALT (GPT) (U/L)**	51.95 ± 2.072	65.11 ± 3.201**	59.69 ± 3.430	59.21 ± 2.941
**CRP (mg/L)**	0.11 ± 0.019	0.143 ± 0.013	0.103 ± 0.028	0.116 ± 0.023
**Glucose (mmol/L)**	5.35 ± 0.146	6.35 ± 0.144***	6.09 ± 0.405	6.15 ± 0.274
**Urea (mmol/L)**	5.857 ± 0.134	5.283 ± 0.288	5.31 ± 0.231	5.022 ± 0.198
**Creatinine (μmol/L)**	29 ± 1.604	30.17 ± 1.272	32.3 ± 1.461	30.67 ± 1.041

The process of aging significantly increased the levels of the total cholesterol, LDL-C and HDL-C, however, the physical exercise had no influence on these parameters. In the aged sedentary animals higher AST, ALT and glucose levels were detected compared to the young controls. To estimate Gaussian distribution, Shapiro-Wilk normality test was used; then, the data were analyzed with unpaired t-test and ordinary one-way ANOVA (or Kruskal-Wallis test). All data are presented as mean ± SEM (** p < 0.01; *** p < 0.001; **** p < 0.0001 compared to the young group). LDL-C: low-density lipoprotein cholesterol; HDL-C: high-density lipoprotein cholesterol; AIP: atherogenic index of plasma; AST: aspartate transaminase; ALT: alanine transaminase; CRP: C-reactive protein.

## RESULTS

### Forced Exercise Mitigates Weight Gain in Aged Rats

**Figure 3.**
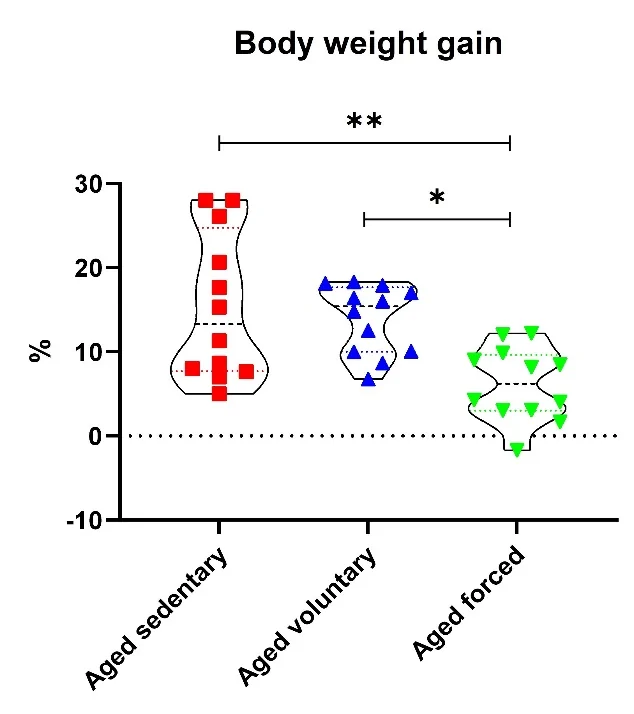
**Weight gain of the aged animals over the 6 months of physical exercise**. Forced exercise reduced weight gain relative to sedentary and voluntary activities. Data are presented as mean ± SEM. All data followed normal distribution, so they were analyzed with ordinary one-way ANOVA followed by Tukey’s post hoc test (* p < 0.05; ** p < 0.01).

The statistical analysis indicated a significant difference between the weight gain of the aged sedentary and the forced exercise groups (p = 0.0038). Moreover, the body weight change of the aged voluntary group was notably higher in comparison to the animals of the forced exercise group (p = 0.018) (Fig. 3).

### Aging Induces Significant Alterations in Lipid Profile

The results of the serum analysis are summarized in Table 1. Total cholesterol, LDL-C and HDL-C levels were significantly elevated in the aged sedentary group, compared to the young controls (p < 0.0001 in all cases). Neither voluntary nor forced running decreased the levels of the above-mentioned parameters, as no significant differences were detected in the lipid profile of the aged animals. Furthermore, no marked changes were observed in the triglyceride content and the calculated AIP value among the experimental groups. Regarding liver and kidney function parameters, only the AST and ALT levels were significantly higher (AST: p < 0.0001; ALT: p = 0.0042) in the aged sedentary group, in comparison to the young rats. Glucose levels showed a similar trend as p = 0.0005 between the young and the old sedentary groups. No perceptible changes were found in the CRP, urea and creatinine levels of the animals.

**Figure 4.**
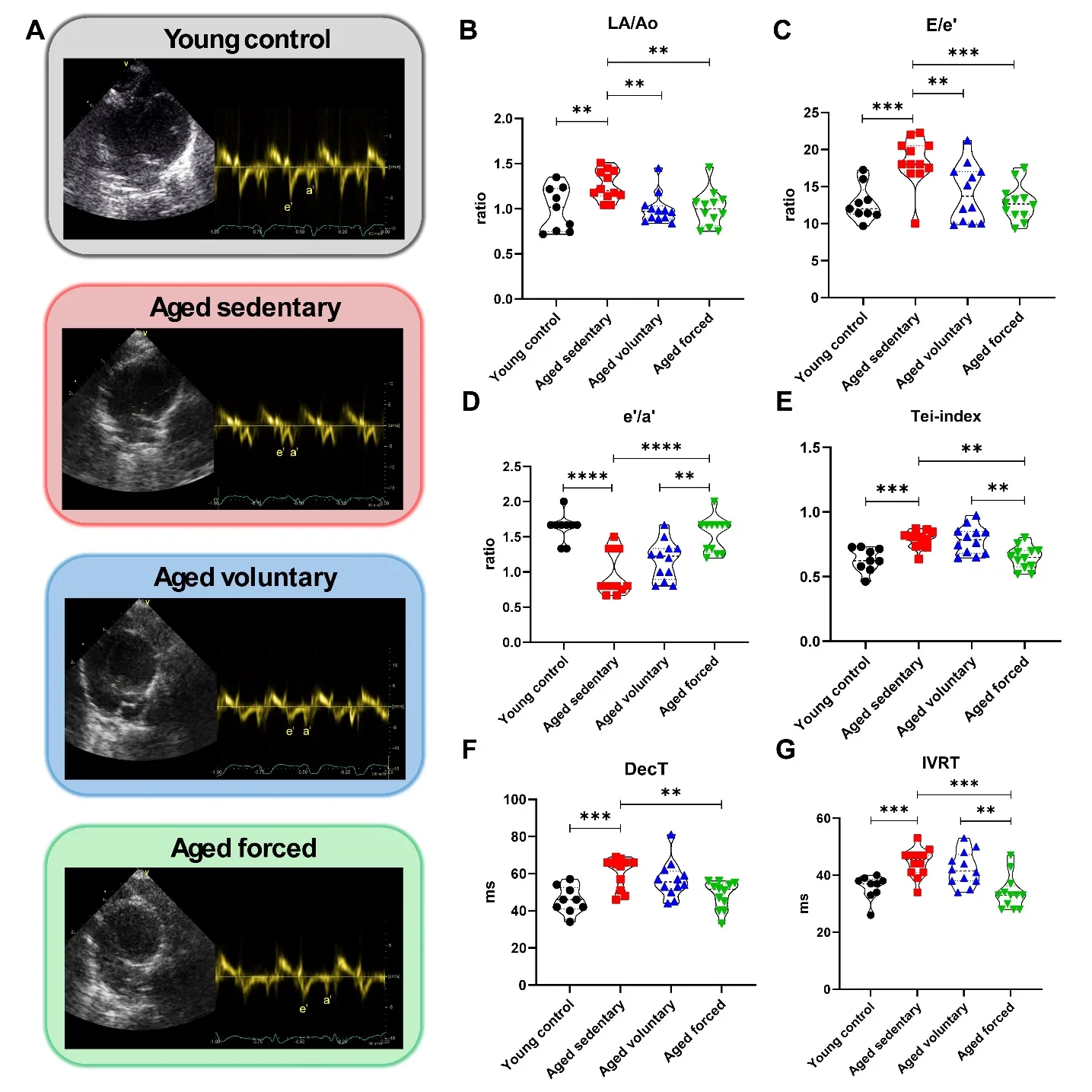
**Echocardiographic parameters representing diastolic function**. (**A**) Representative Tissue Doppler images of the septal mitral annular velocities (e’/a’ ratio). (**B**) The age-related increase in the left atrial-to-aortic root diameter (LA/Ao) ratio was reduced in both exercise groups. (**C**) E/e’ was elevated in the sedentary rats but attenuated in the physically active groups. (**D**) e’/a’ ratio deteriorated in the aged inactive rats and improved in the forced running animals. (**E**) Myocardial performance worsened in the aged sedentary rats but restored in the aged, forced group. (**F**) The aging-associated increase of the deceleration time (DecT) was restored when the rats were subjected to forced exercise. (**G**) Isovolumetric relaxation time (IVRT) increased in the aged sedentary animals but decreased in the forced exercise rats. Data are presented as mean ± SEM. All data followed normal distribution, so they were analyzed with unpaired t-test and ordinary one-way ANOVA followed by Tukey’s post hoc test. (** p < 0.01; *** p < 0.001; **** p < 0.0001).

### Forced Running More Effectively Alleviates Aging-Associated Cardiac Dysfunction Compared to Voluntary Exercise

The outcomes of the echocardiographic measurements are shown in Figure 4. Rats from the aged sedentary group showed marked signs of age-related diastolic dysfunction. Increased LA/Ao and decreased E/A ratios were detected in the physically inactive aged group compared to the young controls (LA/Ao ratio: p = 0.0089; E/A ratio: p < 0.0001). Reduced e’/a’ ratio, as well as lengthened DecT and IVRT were also observed in the aged sedentary rats (e’/a’ ratio: p < 0.0001; DecT: p = 0.0003; IVRT: p = 0.0006 vs young group). Furthermore, significantly elevated Tei-index and E/e’ ratio were noted in the aged animals without exercise versus the young heathy controls (Tei-index: p = 0.0003; E/e’ ratio: p = 0.0004). Regarding the systolic functions, prominent reduction of the EF and FS was observed in the physically inactive aged rats in comparison to the young group (EF: p = 0.001; FS: p = 0.0008). The diastolic function of the left ventricle exhibited marginal improvement across both cohorts of the running subjects, but the changes were more remarkable among the rats from the forced running group. Left atrial enlargement was attenuated in the two physical exercise groups compared to the aged sedentary animals (p = 0.0037 is both cases). In the forced running group, the prolongation of both the DecT and the IVRT were reduced (DecT: p = 0.0032; IVRT: p = 0.0002) and the Tissue Doppler Imaging revealed a significant increase in the e’/a’ ratio (p < 0.0001) versus the aged sedentary rats. Moreover, the physical activity significantly decreased the average E/e’ ratio compared to the results of the aged rats representing inactive lifestyle (Voluntary: p = 0.0083; Forced: p = 0.0006). However, in comparison to the sedentary group, only the regular forced running increased the stroke volume (p < 0.0001) and reduced the Tei-index (p = 0.0013), representing an improvement in the global ventricular function. It is worth noting that there were also significant differences in four echocardiographic parameters of the exercise groups: higher e’/a’ ratio and stroke volume along with decreased IVRT and Tei-index were detected in the forced running rats compared with the voluntary group (e’/a’ ratio: p = 0.0084; stroke volume: p = 0.0001; IVRT: p = 0.0028; Tei-index: p = 0.0018). The values of the heart rate, the ejection fraction and the fractional shortening of the three aging groups were unaffected. Last, but not least, no significant differences were noted between the left ventricle outflow tract (LVOT) parameters of the experimental groups (Table 2).

**Table 2:** Systolic function and LVOT parameters.

Parameter	Young control	Aged sedentary	Aged voluntary	Aged forced
**HR (bpm)**	216.9 ± 6.969	223.7 ± 4.152	216.8 ± 3.958	216.1 ± 9.668
**Stroke volume (µL)**	322.2 ± 34.05	398.3 ± 21.40	450.6 ± 18.95	613.7 ± 26.18^#&^
**EF (%)**	89.00 ± 1.658	78.18 ± 2.097**	79.75 ± 1.931	81.91 ± 2.047
**FS (%)**	55.22 ± 2.548	42.64 ± 1.918***	44.08 ± 1.848	46.64 ± 1.922
**s’ (mm/s)**	0.046 ± 0.002	0.044 ± 0.002	0.047 ± 0.002	0.045 ± 0.003
**MAPSE (mm)**	2.191 ± 0.066	2.327 ± 0.108	2.364 ± 0.107	2.241 ± 0.109
**LVOT Vmax (m/s)**	0.772 ± 0.034	0.792 ± 0.041	0.909 ± 0.028	0.861 ± 0.034
**LVOT Vmean (m/s)**	0.442 ± 0.016	0.493 ± 0.031	0.571 ± 0.022	0.512 ± 0.022
**LVOT maxPG (mmHg)**	2.418 ± 0.214	2.566 ± 0.292	3.235 ± 0.172	3.007 ± 0.238
**LVOT meanPG (mmHg)**	1.011 ± 0.069	1.216 ± 0.142	1.630 ± 0.117	1.323 ± 0.105

The ejection fraction and fractional shortening were significantly reduced in the aged physically inactive rats in comparison to the young counterparts. Stroke volume values were significantly elevated in the forced running rats, compared to both the aged sedentary and voluntary groups. Shapiro-Wilk test was used to estimate Gaussian distribution, then data were analyzed with unpaired t-test and ordinary one-way ANOVA. All data are presented as mean ± SEM (** p < 0.01; *** p < 0.001 compared to young controls; # p < 0.0001 compared to aged sedentary rats; & p < 0.0001 compared to aged voluntary group). HR: heart rate; EF: ejection fraction; FS: fractional shortening; s’: systolic myocardial velocity; MAPSE: mitral annular plane systolic excursion; LVOT: left ventricle outflow tract; Vmax: maximal velocity, Vmean: mean velocity; maxPG: maximal pressure gradient; meanPG: mean pressure gradient.

### Physical Activity Reduces the Fibrosis Localised in the Perivascular Area

**Figure 5.**
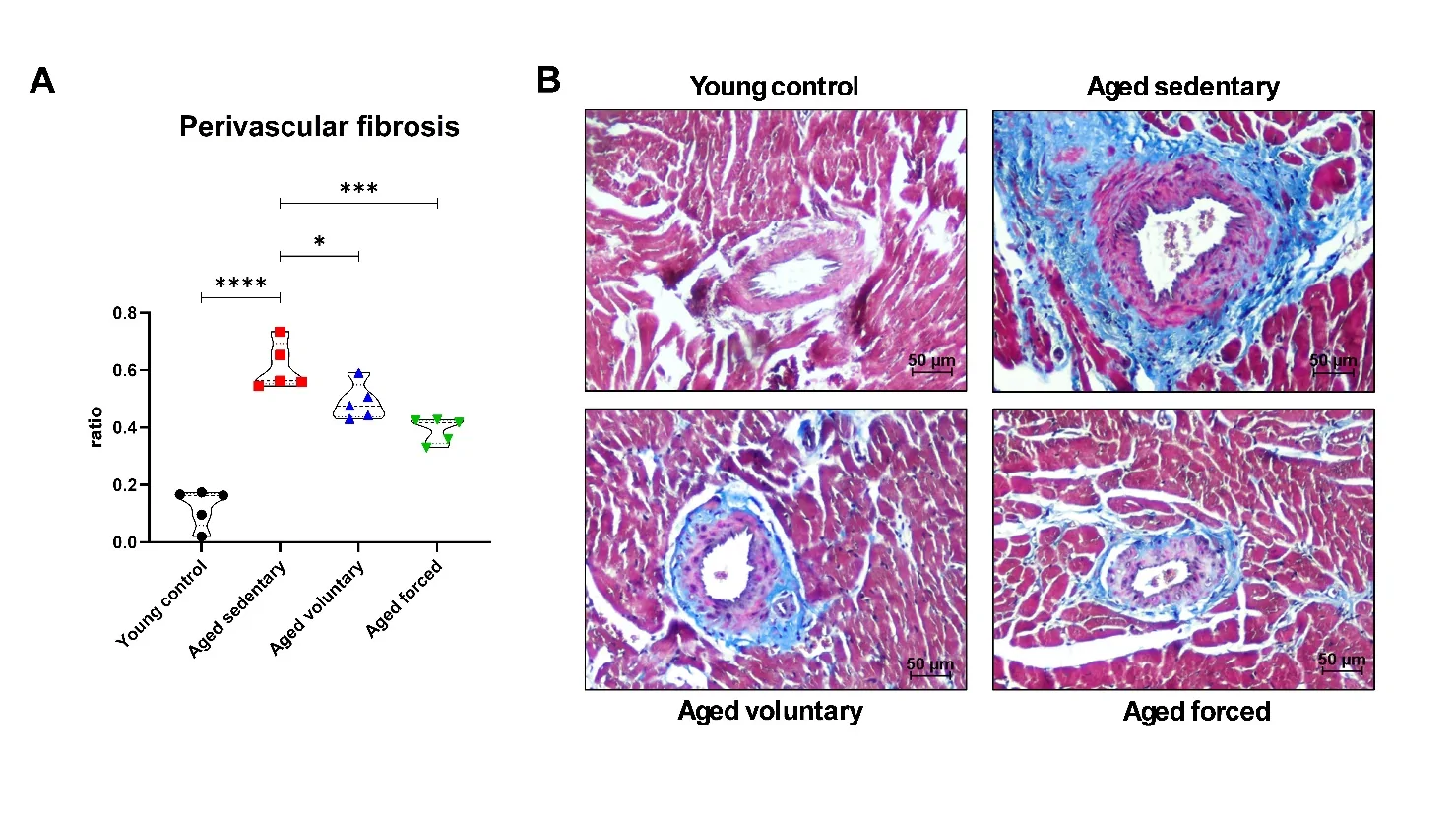
**Histological analysis of the myocardial samples**. (**A**) Graph shows the perivascular fibrosis ratio (PFR) demonstrating both the significant increase in the aged sedentary group compared to the young controls (**** p < 0.0001) and the marked reduction due to the physical exercise (* p < 0.05; *** p < 0.001). Data followed Gaussian distribution. Data were analyzed with unpaired t-test and ordinary one-way ANOVA and are presented as mean ± SEM. (**B**) Representative images of the perivascular accumulation of the fibrotic tissue dyed with blue (magnification: 10 ×).

Aging provoked perivascular fibrosis in the myocardium as the perivascular fibrosis ratio (PFR) was significantly higher in the aged sedentary group compared to the young controls (p < 0.0001). However, the fibrosis was reversed by the physical activity as both the voluntary and the forced exercise significantly decreased the deposition of the connective tissue around the vessels (Voluntary: p = 0.0294; Forced: p = 0.0005 in comparison to the aged sedentary animals). Additionally, no remarkable changes were observed between the two exercise groups (Figure 5).

### Exercise Restores Age-Related Declines in Cardiac PKG, STAT3, Opa1 and ATP Synthase Expression Levels

**Figure 6.**
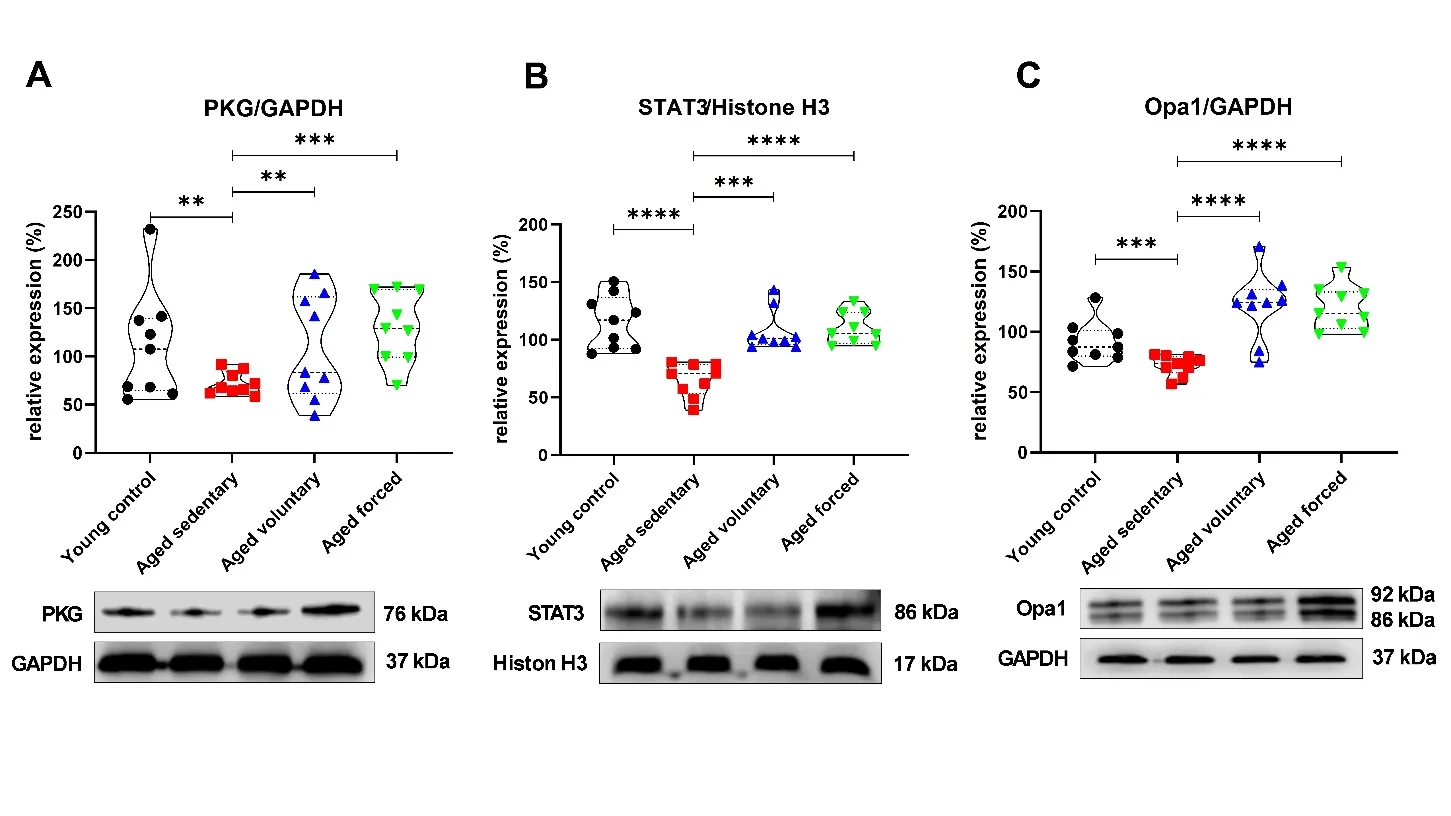
**Graphs demonstrate the results of the quantitative analysis of the density of the bands of the evaluated proteins normalized to GAPDH or Histone H3 (n = 3 per group) along with the representative images**. The aging process significantly reduced the myocardial expression of the PKG (panel (A)), STAT3 (panel (B)) and Opa1 (panel (C)) proteins in comparison to the young controls. Both the voluntary and the forced exercise significantly enhanced the expression of these proteins. All data are presented as mean ± SEM. Shapiro-Wilk normality test was used to estimate Gaussian distribution and then data were analyzed with unpaired t-test and ordinary one-way ANOVA. (** p < 0.01; *** p < 0.001; **** p < 0.0001)

Western blot analysis revealed the age-related downregulation of the PKG-STAT3-Opa1 axis (Figure 6). The myocardial expression of these proteins was decreased in the aged sedentary group in comparison to the young control rats (PKG: p = 0.0092; STAT3: p < 0.0001; Opa1: p = 0.0005). The deterioration of the PKG-STAT3-Opa1 signaling pathway was restored when the aged rats were physically active. In the case of all the three proteins of the axis, the expression levels in the forced and voluntary groups were significantly higher than in the sedentary animals (PKG: sedentary vs voluntary: p = 0.0060, sedentary vs forced: p = 0.0007; STAT3: sedentary vs voluntary: p = 0.0002, sedentary vs forced: p < 0.0001; Opa1: p < 0.0001 in both cases).

**Figure 7.**
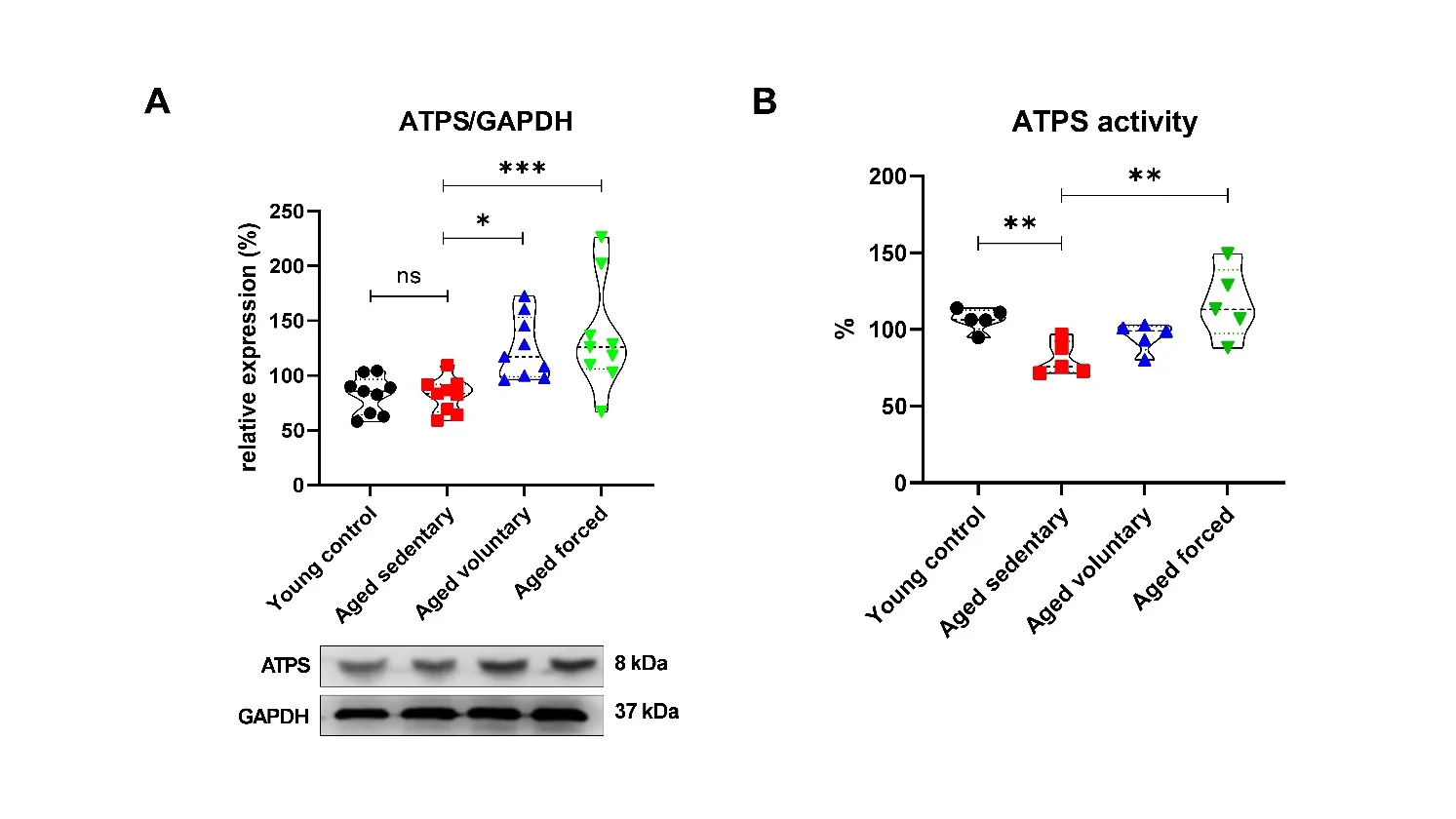
**ATPS expression levels and activity among the experimental groups**. (**A**) Graph shows the statistical analysis and the representative images of the expression of the ATPS normalized to GAPDH (n = 3 per group). Voluntary and forced exercise significantly increased the expression of the ATPS. (**B**) Results of the ATPS activity microplate assay (n = 5 per group). Diminished ATPS activity was detected in the aged inactive group compared to the younger rats, but a marked improvement was determined due to the forced running. All data are presented as mean ± SEM. Shapiro-Wilk normality test was used to estimate Gaussian distribution and then data were analyzed with unpaired t-test and ordinary one-way ANOVA. (* p < 0.05; ** p < 0.01; *** p < 0.001)

Furthermore, the Western blot method was used to exhibit the differences between the expression levels of the ATP synthase in the experimental groups (Figure 7). Interestingly, no marked changes were observed between the healthy young and aged sedentary groups (p = 0.3686). Voluntary and regular forced running significantly elevated the expression of the ATPS compared with the aged inactive group (sedentary vs voluntary: p = 0.0285; sedentary vs forced: p = 0.0002).

Additionally, for all proteins of interest, no significant differences were observed between the two exercise groups (voluntary and forced).

### Forced Running Enhances ATP Synthase Activity

In attempt to confirm the oxidative phosphorylation process of the mitochondria, the activity of the ATP synthase (ATPS) was assessed (Figure 7). A significant decrease was observed in the enzyme activity of the aged sedentary rats compared to the young group (p = 0.0026). The most striking observation to emerge from the data comparison was that forced but not voluntary exercise started at an older age markedly elevated the ATPS activity, compared with the physically inactive aged animals (p = 0.0086). However, voluntary exercise had no significant effect on the activity of the ATPS in aging.

### DISCUSSION

Our present study was designed to examine the cardiovascular effects of long-term voluntary and forced exercise in an aging rat model.

The serum analysis results indicated significant deterioration in the lipid profile, with increased total cholesterol, LDL-C and HDL-C levels in the older groups. These findings are advocated by previous scientific literature describing the condition of senescence-associated dyslipidaemia [27, 28]. However, neither voluntary nor forced physical activity reduced age-induced hypercholesterolemia. An alternative explanation for this result is that aging may lead to changes in lipid metabolism, resulting in decreased responsiveness to lifestyle interventions [29]. Here, it is important to note that the rat animal model is not appropriate for monitoring dyslipidemia [30]. Additionally, our morphometric results are directly in line with previous research, showing significantly less weight gain in the forced exercise group compared to both the aged inactive and voluntary exercise groups [31]. This finding highlights that structured, forced physical activity might provide superior benefits for weight management compared to voluntary exercise in aged rats.

Regarding cardiovascular status, our initial goal was to identify functional and structural cardiac abnormalities that arise solely due to aging, independent of any pathological conditions. At the endpoint of the study, the echocardiographic measurements demonstrated decreased E/A and e’/a’ ratios and lengthened DecT and IVRT in the aged sedentary rats, indicative for impaired myocardial relaxation. Moreover, elevated left ventricular filling pressure was also estimated, due to the higher E/e’ ratio [32]. These changes, together with the marked LA enlargement, provide conclusive evidence to establish the diagnosis of diastolic dysfunction in the 2-year-old physically inactive rats [33]. This age-related deterioration of the diastolic function was also shown by Rowe et al, who determined the cardiac changes between 3-month-old and 24-month-old rats [34]. Furthermore, considering the absence of fibrosis in the young healthy group, our results suggest that the significant collagen deposition around the vessel walls observed in the sedentary group was age-related. The consequences of fibrosis, such as increased ventricular stiffness causing delayed relaxation and impaired LV filling, have also been detected through cardiac ultrasonography [35]. Our histological findings are consistent with the results seen in the study of Reed et al, as they showed that the deterioration of the diastolic function was accompanied by enhanced cardiac fibrosis in a senescence-accelerated mouse model [36]. Moreover, Horn and Trafford demonstrated that there is a strong relationship between perivascular fibrosis and cardiac aging as well [37]. To be more precise, the accumulation of connective tissue surrounding the vessels in the myocardium leads to coronary microvascular dysfunction (CMD), which contributes to accelerated tissue senescence [38].

The diastolic performance showed some improvement in both exercise groups, including reduced LV filling pressure and LA enlargement characterized by decreased E/e’ and LA/Ao ratios. It is noteworthy that further echocardiographic parameters suggesting better myocardial relaxation such as reduced IVRT and DecT along with elevated e’/a’ ratio were restored only in the rats subjected to the forced running regimen [39, 40]. The attenuating effect of the physical activity on age-associated diastolic dysfunction has also been shown by Brenner et al, who studied the effects of 12 weeks of treadmill running on 24-month-old rats [41]. Regarding the Tei-index defining the global LV systolic and diastolic performance, our result is consistent with what has been found in the investigation by Cho et al, as they also detected decreased Tei-index in a mouse model of late-in-life treadmill running [42]. In terms of our echocardiographic and histological results, we assume that forced physical activity may protect against the age-induced decline in the diastolic performance by reversing perivascular fibrosis and enhancing microvascular function, thus improving myocardial relaxation and coronary perfusion in old rats. Similar outcomes were reported by Hotta et al, who subjected 20-month-old rats to 10-12 weeks of physical activity and concluded that late-life exercise can reverse diastolic and microvascular endothelial dysfunction [43].

Concerning the molecular mechanisms, a study by Chang et al has highlighted the PKG-STAT3-Opa1 pathway, describing it as a potent promoter of mitochondrial fusion in diabetic cardiomyopathy [14]. Since the imbalance between the mitochondrial fusion and fission dynamics is one crucial reason of cardiac senescence, Western blot technique was carried out to identify the expression of the proteins of the PKG-STAT3-Opa1 axis. In the present study, the reduced myocardial PKG expression in the aged physically inactive rats further supports the diagnosis of the abnormal diastolic function [44, 45]. This result is consistent with findings from previous research conducted using different models of diastolic dysfunction [46, 47]. Concerning the STAT3 protein, lower levels of the transcription factor indicate diminished cardioprotection due to the aging process in the sedentary animals [48]. Hilfiker-Kleiner et al concluded the fact that reduced STAT3 expression and activation contribute to the progression of heart failure by playing a crucial role in myocardial stress adaptation and proper cardiac function, confirming our results [49]. Additionally, the reduced expression of the transcriptional target Opa1 in the hearts of the aged sedentary group suggests impaired mitochondrial fusion, leading to abnormal mitochondrial dynamics and contributing to heart failure [50, 51]. This finding is directly in line with the study of Chen et al, who detected mitochondrial dysfunction along with reduced ATP levels and increased ROS production in heterozygous Opa1^+/-^ mice [52]. In summary, the current study revealed the downregulation of the PKG-STAT3-Opa1 pathway as a consequence of aging, evidenced by lower expression levels of the proteins in the left ventricle samples of the aged sedentary rats in comparison to the young control animals.

A large number of studies in the scientific literature have examined the health benefits of physical activity, however, to identify the underlying molecular mechanisms by which exercise promotes multiple defensive changes in the human body is still an unexplored area of medical research [53]. To the best of our knowledge, this is the first experiment that reveals the exercise-associated upregulation of the PKG-STAT3-Opa1 axis, thus demonstrating a novel signaling pathway associated with cardioprotection in aging. Several studies have exhibited that PKG induces some protective signaling in the heart in a number of pathological conditions. In this investigation the observed enhanced PKG expression due to the physical activity and its hypothesized cardioprotective effect is in consonance with the research by Heerebeek et al, who concluded the correction of the myocardial PKG level in the treatment of diastolic heart failure [54]. The increased STAT3 expression that we detected in the physically active rats was supported by recent evidence noting that in contrast to oncogenesis, the activation of STAT3 is required in mediating cardioprotective processes [55]. Furthermore, accumulating evidence demonstrates that restoring the abnormal mitochondrial dynamics by promoting the Opa1-mediated mitochondrial fusion is one of the key approaches that can enhance tissue function. In terms of diabetic cardiomyopathy, punicalagin and paeonol are potent mitochondrial fusion promoters by regulating STAT3 and Opa1 levels [13, 56]. Moreover, κ-opioid receptor activation induces mitochondrial fusion resulting in enhanced resistance to ischemia and reperfusion injury via the myocardial STAT3-Opa1 pathway [16].

Furthermore, the expression and activity of ATP synthase were also examined as part of the molecular pathway study. The microplate assay showed a marked decline in the ATPS activity in the physically inactive aged animals. The reduced mitochondrial ATPS activity is indicative for impaired ATP production along with an excessive amount of ROS resulting in oxidative stress that can be speculated to contribute to the age-related cardiac dysfunction [57]. This theory is supported by scientific literature indicating that mitochondrial dysfunction leads to cardiac energy deprivation and exacerbates heart failure [58].

Based on the results of the ATPS activity assay, it can be hypothesized that the Opa1-mediated fusion is followed by increased ATPS activity due to forced but not voluntary exercise in aging rats. These outcomes are supported by previous data showing that activating the Opa1-mediated mitochondrial fusion machinery leads to increased OXPHOS and ATP production along with decreased ROS accumulation [50, 59]. Moreover, Opa1 has been shown to regulate mitochondrial cristae architecture, thus it maintains proper cristae structure that ensures ideal set-up for ATP generation [60].

This research still has some limitations. It is important to note that while rats are commonly used models for representing and studying human aging and physical activity, this model is not able to fully replicate the changes in the human body caused by aging or exercise. Secondly, our study was conducted exclusively with male experimental animals; however, incorporating both sexes into the study design would have resulted in more reliable and reproducible findings.

In conclusion, our study represents the first exploration of whether voluntary or forced physical activity can improve diastolic function via the cardiac PKG-STAT3-Opa1 pathway and the myocardial ATP synthase activity related to aging. Our findings indicate that only the forced activity alleviated effectively the age-related cardiac dysfunction and enhanced the mitochondrial function. Although further research is required, this study offers new insights into how exercise affects the aging heart, potentially uncovering new targets for treating age-related cardiac dysfunction.

## Conceptualization

R.S., B.B., B.P.-T., A.Sz. and B.J.; methodology, R.S., D.P., M.B., B.V. and P.A.; validation, R.G., B.V and P.A.; investigation, R.S., B.J., D.P. and M.B.; resources, B.J. and Z.Sz..; writing—original draft preparation, R.S. and R.G.; writing—review and editing, B.J.; visualization, R.S. and S.S.; supervision, Z.Sz. and B.J.; project administration, Z.Sz.; funding acquisition, Z.Sz. and B.J.

All authors have read and agreed to the published version of the manuscript.

## Data and materials availability

The data that support the findings of this study are available from the corresponding author upon reasonable request.
